# Antisense oligonucleotides targeting hepatic angiotensinogen reduce atherosclerosis and liver steatosis in hypercholesterolemic mice

**DOI:** 10.36922/gtm.288

**Published:** 2023-02-24

**Authors:** Dien Ye, Congqing Wu, Lei Cai, Deborah A. Howatt, Ching-Ling Liang, Yuriko Katsumata, Adam E. Mullick, Ryan E. Temel, A.H. Jan Danser, Alan Daugherty, Hong S. Lu

**Affiliations:** 1 Saha Cardiovascular Research Center, University of Kentucky, Lexington, KY, USA; 2 Division of Vascular Medicine and Pharmacology, Department of Internal Medicine, Erasmus MC, Rotterdam, Netherlands; 3 Saha Aortic Center, University of Kentucky, Lexington, KY, USA; 4 Department of Surgery, University of Kentucky, Lexington, KY, USA; 5 Department of Microbiology, Immunology, and Molecular Genetics, University of Kentucky, Lexington, KY, USA; 6 Department of Biostatistics, College of Public Health, University of Kentucky, Lexington, KY, USA; 7 Sanders-Brown Center on Aging, University of Kentucky, Lexington, KY, USA; 8 Ionis Pharmaceuticals, Inc., Carlsbad, CA, USA; 9 Department of Physiology, University of Kentucky, Lexington, KY, USA

**Keywords:** Angiotensinogen, Antisense oligonucleotides, Blood pressure, Liver steatosis, Atherosclerosis

## Abstract

Hepatocyte-derived angiotensinogen (AGT) is the precursor of angiotensin II (AngII). We determined the effects of hepatocyte-specific (*N*-acetylgalactosamine-conjugated) antisense oligonucleotides targeting AGT (GalNAc AGT ASO) on AngII-mediated blood pressure (BP) regulation and atherosclerosis and compared its effects with losartan, an AngII type 1 (AT1) receptor blocker, in hypercholesterolemic mice. Eight-week-old male low-density lipoprotein (LDL) receptor deficient mice were administered vehicle or GalNAc AGT ASO (1, 2.5, or 5 mg/kg) subcutaneously beginning 2 weeks before the initiation of Western diet feeding. All mice were fed Western diet for 12 weeks. Their systolic BP was monitored by the tail-cuff technique, and the atherosclerotic lesion area was measured by an *en face* method. Although the effects of all 3 doses of GalNAc AGT ASO on plasma AGT concentrations were similar, GalNAc AGT ASO reduced BP and atherosclerotic lesion size in a dose-dependent manner. Subsequently, we compared the effects of GalNAc AGT ASO (5 mg/kg) with losartan (15 mg/kg/day). Compared to losartan, GalNAc AGT ASO led to more profound increases in plasma renin and reduction in BP but had similar effects on atherosclerosis. Remarkably, GalNAc AGT ASO also reduced liver steatosis, which was not observed in losartan-treated mice. In conclusion, the BP increase and atherosclerosis development in hypercholesterolemic mice are dependent on AngII generated from hepatic AGT. Deleting hepatic AGT improves diet-induced liver steatosis, and this occurs in an AT1 receptor-independent manner.

## 1. Introduction

Angiotensinogen (AGT) is cleaved by renin to generate angiotensin (Ang) I and des(AngI)AGT^[1,2]^. Subsequently, AngI is cleaved by angiotensin-converting enzyme (ACE) to release AngII. AngII, through binding to AngII type 1 (AT1) receptor, contributes to blood pressure (BP) regulation and atherosclerosis^[3–5]^. In previous studies, the pharmacological inhibition of renin, ACE, or AT1 receptor reduced BP and atherosclerosis in low-density lipoprotein (LDL) receptor^−/−^ mice fed a Western diet^[3–7]^. Whole-body reduction of AGT, hepatocyte-specific AGT deficiency (hepAGT^−/−^), and global AGT antisense oligonucleotides (ASO) exerted similar effects on BP and atherosclerosis in LDL receptor^−/−^ mice^[8]^.

While the AngII-dependent functions of AGT have been well-documented^[1,9]^, the function of the des(AngI) AGT portion of AGT has not been extensively studied. Recently, the biological function of des(AngI)AGT has been investigated by infecting hepAGT^−/−^ mice with an adeno-associated virus (AAV) encoding des(AngI)AGT. The expression of des(AngI)AGT in hepAGT^−/−^ mice increased Western diet-induced body weight gain and liver steatosis but had no effects on BP and atherosclerosis^[8]^. These data support the notion that the effects of AGT deletion on BP and atherosclerosis are AngII-dependent, whereas des(AngI)AGT has effects on metabolic disorders in an AngII-independent manner.

Although hepatocytes are the major source of plasma AGT, its synthesis has also been reported at non-hepatic sites^[10–13]^. The degree to which non-hepatic sources of AGT contribute to the above effects remains unknown. Studies have shown that adipocyte- or macrophage-derived AGT deficiency has marginal or no effect on BP and atherosclerosis^[8,10,11]^. To verify the importance of hepatic AGT from a pharmacological approach, we targeted hepatocyte AGT mRNA, making use of *N*-acetylgalactosamine (GalNAc)-conjugated AGT ASO^[14]^, in LDL receptor^−/−^ mice fed a Western diet for 12 weeks. A comparison was made versus losartan, a classic AT1 receptor blocker, to distinguish AngII-dependent and independent effects.

## 2. Materials and methods

### 2.1. Animals

**Figure 1.**
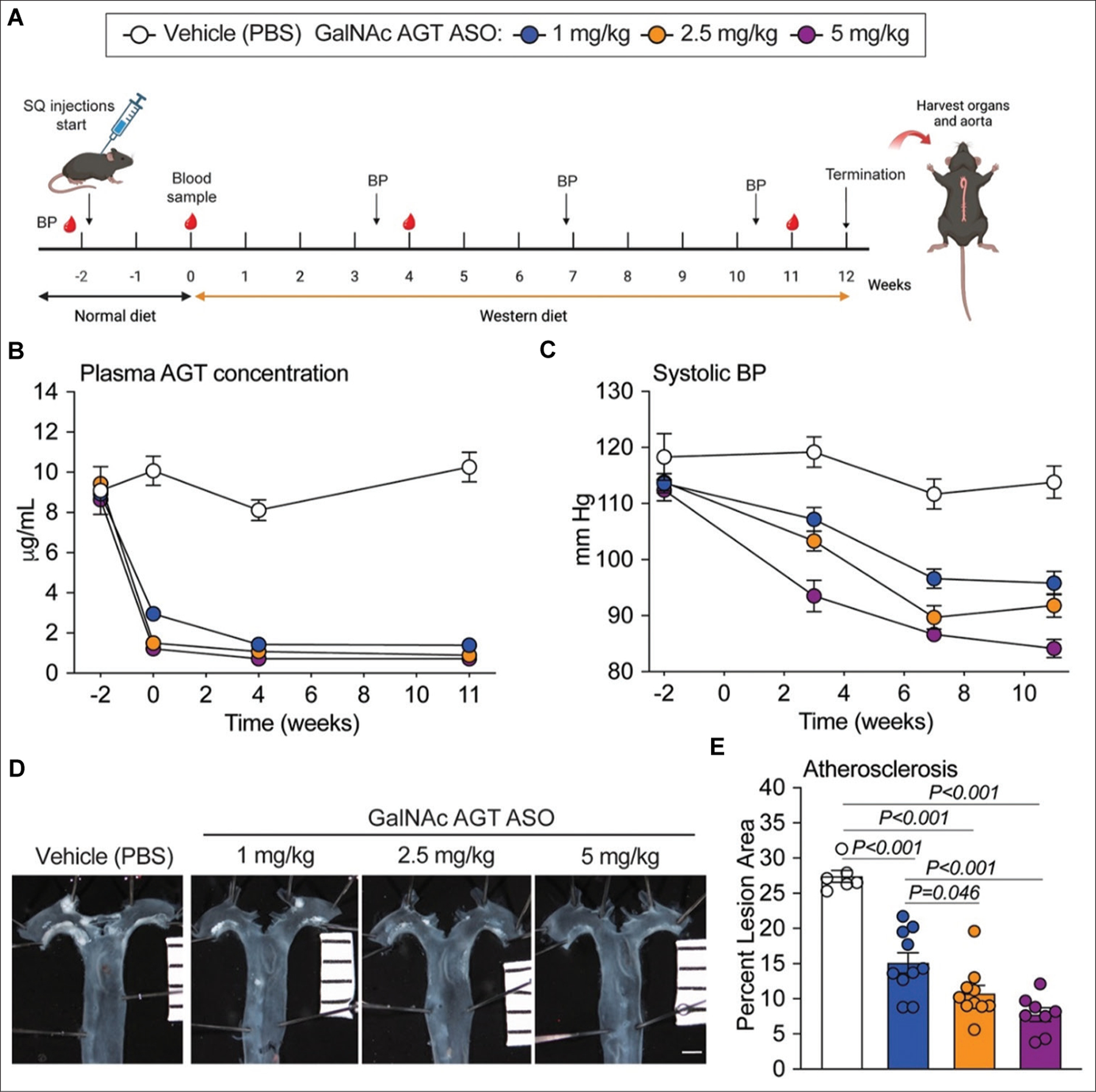
Antisense oligonucleotides (ASO) targeting hepatic angiotensinogen (AGT) reduced plasma AGT concentrations, systolic blood pressure, and atherosclerotic lesion area in the thoracic aorta. (A) Experimental protocol. (B) Plasma AGT concentrations on weeks −2, 0, 4, and 11 during ASO administration. A piecewise mixed-effect model was fitted to examine slopes for distinct time points (−2 to 0 and 0 to 11 weeks). Doses 1, 2.5, and 5 mg/kg led to profound reductions in plasma AGT concentrations compared to week −2; *P* < 0.001. (C) Mouse systolic blood pressure (BP) on weeks −2, 3, 7, and 11. A mixed-effect model was used to compare slopes among the four groups. Doses 1, 2.5, and 5 mg/kg led to significant reductions in systolic BP compared to week −2; *P* < 0.001. (D) *En face* aorta images, scale bar = 1 mm. (E) Atherosclerotic lesion area of the thoracic aorta normalized by total intimal surface area; N = 6–10 per group. GalNAc AGT ASO: N-acetylgalactosamine-conjugated antisense oligonucleotides targeting angiotensinogen; PBS: Phosphate-buffered saline; SQ: Subcutaneous.

Male LDL receptor deficient mice (LDL receptor^−/−^, strain # 002207) were purchased from The Jackson Laboratory (Table S1). Eight-week-old male LDL receptor^−/−^ mice (Table S2) were subcutaneously injected with phosphate-buffered saline (PBS; vehicle) or GalNAc AGT ASO (1, 2.5, or 5 mg/kg) beginning 2 weeks before Western diet feeding (diet # TD.88137, Envigo). Week −2 represents the start of subcutaneous injection of either PBS or GalNAc AGT ASO, while week 0 represents the 1^st^ week of Western diet feeding (Figure 1A). On week −2, the mice were injected with vehicle or any of the 3 doses of GalNAc AGT ASO on days 1, 3, and 5, and then the 1^st^ day of week −1. Two weeks after the initiation of subcutaneous injections (week 0), the mice were fed Western diet for 12 weeks, while vehicle or GalNAc AGT ASO was injected once every week. GalNAc AGT ASO was provided by Ionis Pharmaceuticals Inc. (Carlsbad, CA, USA).

We then tested the rapidity of GalNAc AGT ASO reduction in plasma AGT concentrations. Male C57BL/6J mice (~8 weeks old) were injected with GalNAc AGT ASO 10 mg/kg once (Table S3), and their plasma samples were collected sequentially for 10 days. Subsequently, GalNAc AGT ASO and losartan were compared. Eight-week-old male LDL receptor^−/−^ mice were randomly assigned to three groups (Table S4). The mice in Group 1 (vehicle) were subcutaneously injected with PBS (on days 1 and 3, then once every week) and implanted a pump to deliver water subcutaneously; mice in Group 2 (GalNAc AGT ASO) were subcutaneously injected with GalNAc AGT ASO (5 mg/kg on days 1 and 3, then once every week) and implanted a pump to deliver water subcutaneously; mice in Group 3 (losartan) were subcutaneously injected with PBS (on days 1 and 3, then once every week) and implanted a pump to deliver losartan (15 mg/kg/day; Cat # 61188–100 mg, Millipore Sigma). The mini osmotic pump ALZET Model 2006 (Durect Corp.) was used to deliver water or losartan subcutaneously. The first mini osmotic pumps were replaced by second ones after 6 weeks of infusion. Week −1 represents the start of subcutaneous injection, while week 0 represents the first week of Western diet feeding. On week −1, the mice were injected with PBS or GalNAc AGT ASO on days 1 and 3, and then once each week. Mini osmotic pumps were implanted on day 5 of week −1 to deliver either water or losartan. One week after the initiation of subcutaneous injections (week 0), the mice were fed Western diet for 12 weeks.

All animal experiments (Table S5 and S6) reported in this article were performed with the approval of the University of Kentucky Institutional Animal Care and Use Committee (IACUC protocol number 2018–2968 or 2015–2050).

### 2.2. Systolic blood pressure

Systolic BP was measured in conscious mice using a non-invasive tail-cuff system (BP-2000, Visitech Systems) following our standard protocol^[15]^ and was calculated based on 20 measurements per mouse per day for 3 consecutive days. The mean systolic BP of each mouse from the 3-day measurements was used for data analysis.

### 2.3. Plasma profiles

Mouse blood was collected in the presence of ethylenediaminetetraacetic acid (EDTA; final concentration: 1.8 mg/mL) through submandibular bleeding during the study. At termination, blood was collected through cardiac bleeding through the right ventricle. Plasma AGT concentrations were measured using a mouse AGT enzyme-linked immunosorbent assay (ELISA) kit (Cat # 245718, Abcam), which detects both intact AGT and des(AngI)AGT, thereby measuring total AGT. Plasma renin concentrations in mice were measured by an enzyme-kinetic assay using an AngI ELISA kit (Cat #: IB59131, IBL-America) after being incubated with exogenous recombinant mouse AGT for 1 h.

Plasma alanine aminotransferase (ALT) and aspartate aminotransferase (AST) were measured on the Olympus AU400 clinical analyzer (Olympus, Center Valley, PA) using L-Type ALT.J2 and L-Type AST.J2 reagents and calibrators (Fujifilm Healthcare) in Ionis in a blinded manner.

### 2.4. Quantification of atherosclerosis

After euthanasia, mouse aortas were dissected and fixed in 10% neutrally buffered formalin overnight. Subsequently, adventitial tissues were removed, and the intimal surface was exposed by a longitudinal cut and pinned on a black rubber surface. Images of *en face* aortas were taken using a digital camera (Nikon Digital Sight DS-Ri1), with a ruler for calibration.

An *en face* method was used to measure atherosclerotic lesions on the intimal surface of the aorta in accord with the American Heart Association (AHA) statement and as detailed in our standard protocol^[16,17]^. Atherosclerotic lesions were traced manually from the ascending aorta to the proximal part of the descending thoracic aorta (1 mm distal from the orifice of the left subclavian artery) using Nikon NIS-Elements software (NIS-Elements AR 5.11.00.) under a dissecting microscope.

### 2.5. Histology

At termination, a piece of each liver sample was fixed in paraformaldehyde (4% wt/vol) overnight and then embedded in paraffin. Five-micron sections were used for hematoxylin and eosin (H&E) staining. In addition, a piece of liver (fresh frozen) was embedded in optimal cutting temperature compound (OCT; Cat # 14–373-65, Fisher Scientific), sectioned using a cryostat (Leica CM 1850, Leica) at 10 μm/section, and stained with Oil Red O to visualize neutral lipid accumulation.

### 2.6. Quantification of liver steatosis

Liver weights were recorded at termination. A small piece of liver was snap-frozen in liquid nitrogen. Liver lipids were extracted, solubilized with Triton X-100, and quantified using enzymatic assays kits for total cholesterol (Cat # 23–66-201, Pointe Scientific Cholesterol reagent), and triglycerides (Regents 1 and 2: Cat # 994–02891 and Cat # 990–0299, Fujifilm Healthcare)^[18]^.

### 2.7. RNA isolation and quantitative polymerase chain reaction (PCR)

Total RNA was extracted from liver and kidney samples using a commercial kit (Cat # AS1280, Promega) and the automated Maxwell^®^ RSC 48 Instrument (Promega). To quantify mRNA abundance, total RNA was reversely transcribed with iScript^™^ cDNA Synthesis kit (Cat # 170–8891, Bio-Rad), and quantitative PCR (qPCR) was performed using the TaqMan^™^ Fast Advances Master Mixes kit (Cat # A44359, Thermo Fisher Scientific) on a Bio-Rad CFX96 cycler. TaqMan assay primers: *Agt* (ID: Mm00599662_m1), *Ren1* (ID: Mm02342889_g1), *Gapdh* (ID: Mm99999915_g1), *Actb* (ID: Mm01205647_g1), and *Ppia* (ID: Mm02342429_g1). Data were analyzed using the ΔΔCt method and normalized with the geometric mean of the three reference genes: *Gapdh, Actb,* and *Ppia*.

### 2.8. Statistical analysis

There were two types of data in this study: non-repeated measures after termination, and repeated measures during the study. Prism v9 (GraphPad Software Inc., La Jolla) was used for non-repeated measures, while R version 4.2.1 was used for repeated measures. Before analyzing non-repeated measures, normality and homogeneous variance assumptions were tested with Shapiro–Wilk and Brown-Forsythe tests, respectively. Since these assumptions were satisfied, all non-repeated data were analyzed using one-way analysis of variance (ANOVA) to compare means among three or more groups, followed by the Sidak *post hoc* test. For repeated measures, mixed-effect models with inverse-variance weights were used with random intercept and slope for time. A piecewise model was fitted to estimate separate slopes for distinct time points (−2 to 0 and 0 to 11 weeks) in plasma AGT concentrations. The mixed-effect models were run using the lme function in the nlme R package. Data of non-repeated measures were represented as individual data points and mean ± standard error of the mean (SEM). *P* < 0.05 or Bonferroni-corrected *P <* 0.05 was considered statistically significant.

## 3. Results

### 3.1. *N*-acetylgalactosamine-conjugated antisense oligonucleotides targeting angiotensinogen reduced blood pressure, atherosclerosis, and Western diet-induced liver steatosis

In the vehicle group, plasma AGT remained unaltered (Figure 1B) in mice fed either normal laboratory diet (from week −2 to week 0) or Western diet (from week 0 through week 12). GalNAc AGT ASO significantly reduced plasma AGT concentrations in mice when they were fed normal laboratory diet; Western diet did not alter this outcome. All three doses of GalNAc AGT ASO yielded the same degree of AGT lowering, maximally reducing plasma AGT by ~90%. GalNAc AGT ASO reduced systolic BP in a dose-dependent manner versus vehicle (Figure 1C), with the effect at 5 mg/kg being significantly larger than that at 1 mg/kg (*P* < 0.001). Similarly, a dose-dependent reduction in atherosclerotic lesion size was observed (Figure 1D and E).

**Figure 2.**
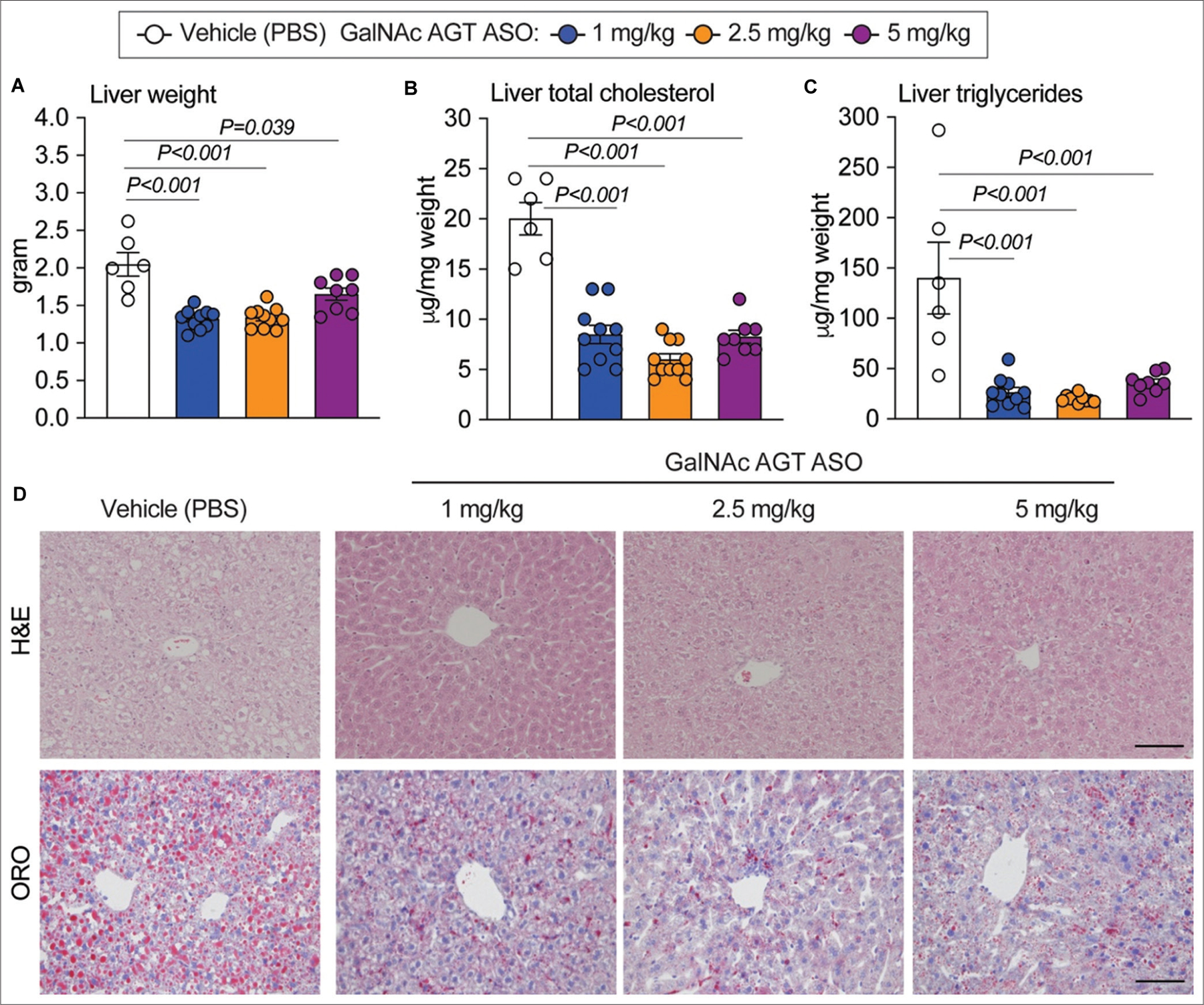
Antisense oligonucleotides targeting hepatic angiotensinogen attenuated liver steatosis. (A) Liver weight at termination. (B) Liver total cholesterol concentration (normalized by liver weight). (C) Liver triglycerides concentration (normalized by liver weight). (D) Hematoxylin and eosin (H and E) staining (paraffin-embedded sections) and Oil Red O (ORO) staining (fresh-frozen sections) of liver sections; scale bar = 200 μm. N = 6 – 10 per group. GalNAc AGT ASO: N-acetylgalactosamine-conjugated antisense oligonucleotides targeting angiotensinogen; PBS: Phosphate-buffered saline.

LDL receptor^−/−^ mice fed a Western diet developed liver steatosis with increased liver weight and liver cholesterol and triglyceride content^[8,19–22]^. The results obtained in this study (Figure 2A–C) confirmed this outcome. GalNAc AGT ASO administration reduced liver weight and liver total cholesterol and triglyceride content (Figures 2A–C) in a dose-independent manner. H&E staining and Oil Red O staining revealed diminished neutral lipid accumulation in mice administered GalNAc AGT ASO at all doses compared to the vehicle group (Figure 2D).

### 3.2. Comparisons between *N*-acetylgalactosamine-conjugated antisense oligonucleotides targeting angiotensinogen and losartan

**Figure 3.**
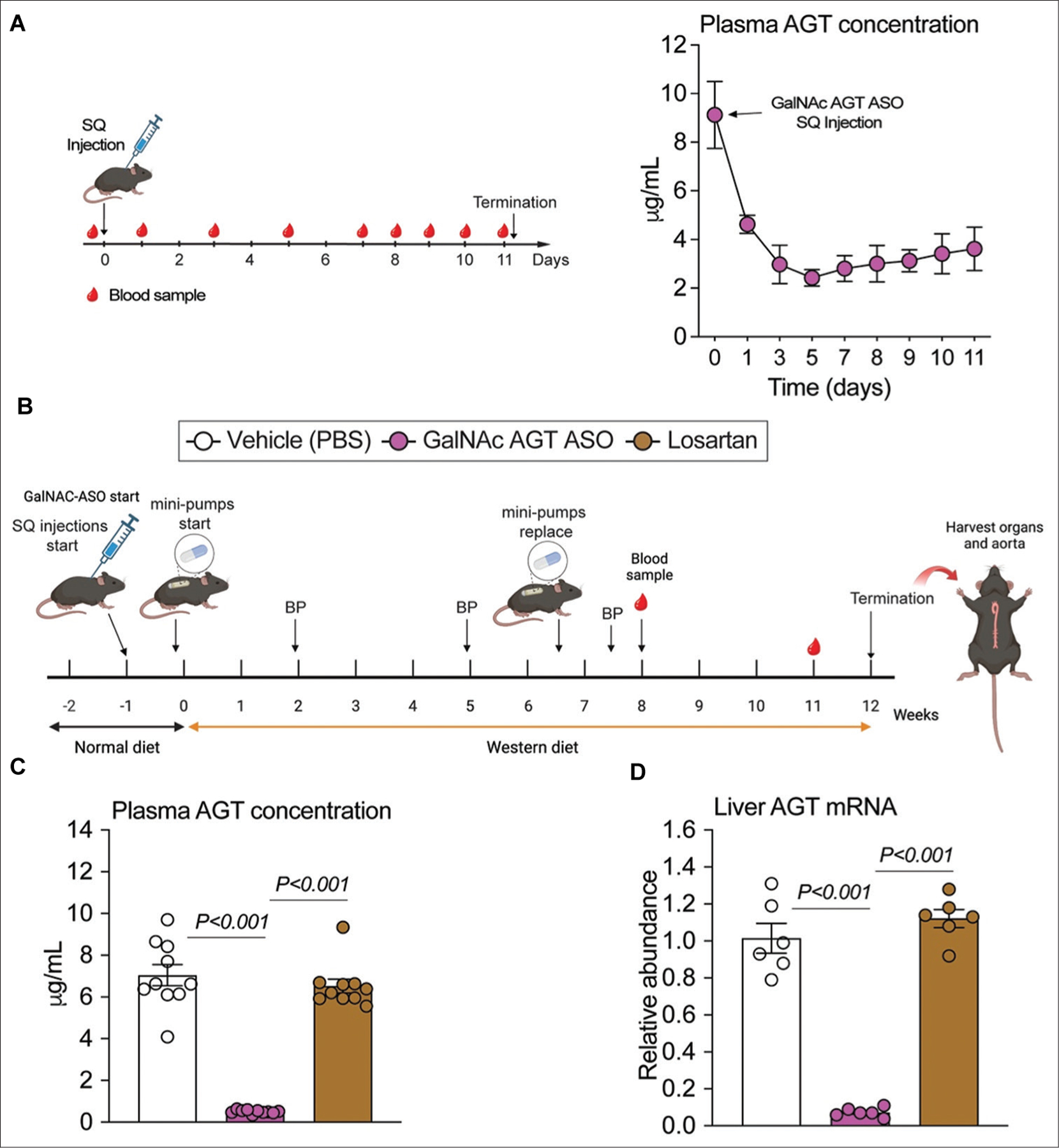
Effects of hepatocyte-specific antisense oligonucleotides of angiotensinogen (AGT) versus losartan on AGT protein and mRNA. (A) Experimental protocol for a single dose of GalNAc AGT ASO injection in male C57BL/6J mice. Plasma AGT concentrations were measured using an enzyme-linked immunosorbent assay (ELISA) kit; N = 5. (B) Experimental protocol of GalNAc AGT ASO and losartan comparisons. (C) Plasma AGT concentrations; N = 10 per group. (D) Quantitative polymerase chain reaction of liver AGT mRNA; N = 6 per group. GalNAc AGT ASO: N-acetylgalactosamine-conjugated antisense oligonucleotides targeting angiotensinogen; PBS: Phosphate-buffered saline; SQ: Subcutaneous.

**Figure 4.**
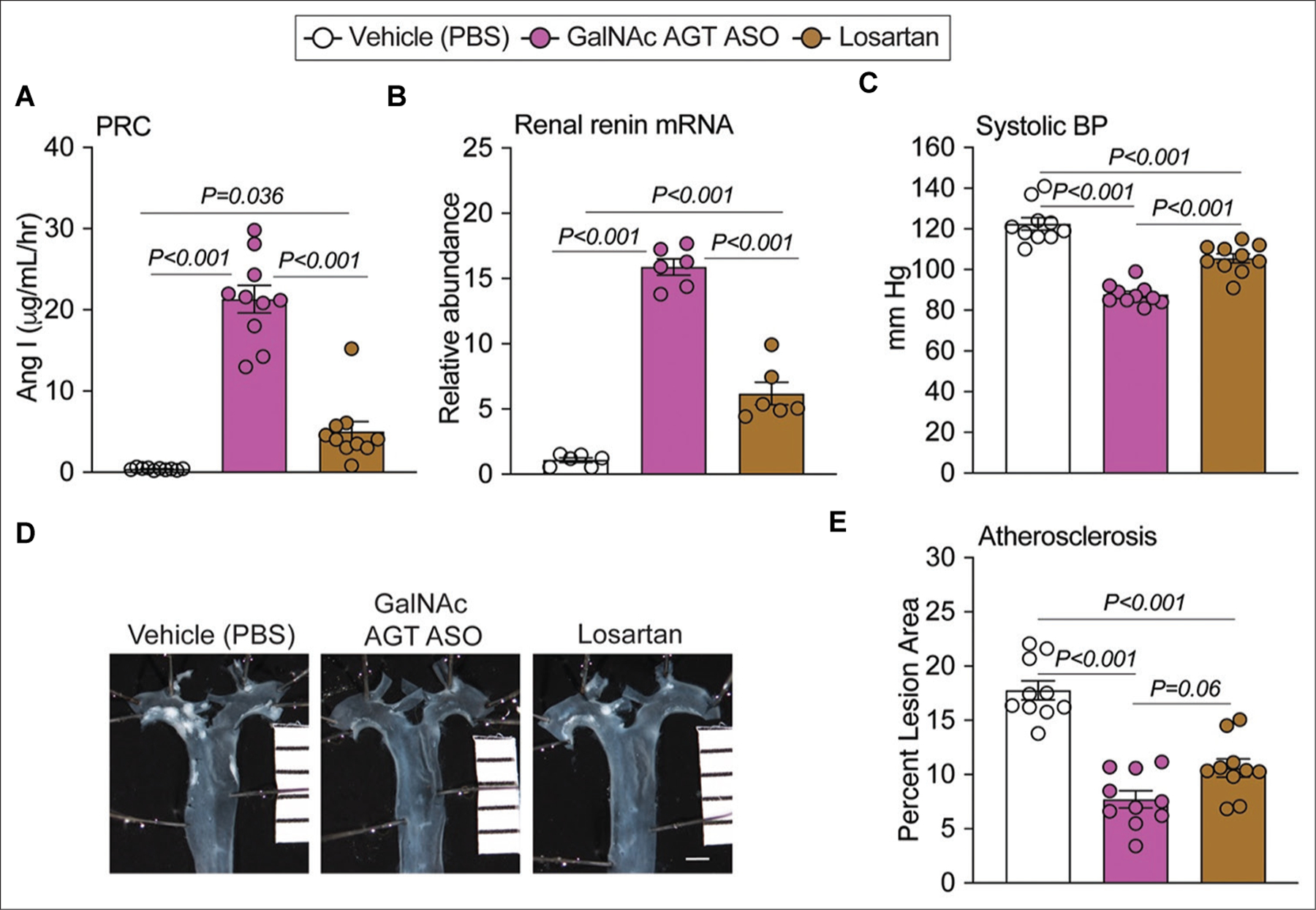
Effects of hepatocyte-specific antisense oligonucleotides of angiotensinogen versus losartan on renin, systolic blood pressure, and atherosclerotic lesion size. (A) Plasma renin concentration (PRC); N = 10 per group. (B) Renal renin mRNA abundance by quantitative polymerase chain reaction; N = 6 per group. (C) Systolic blood pressure (BP) at week 7. (D) *En face* aortic images; scale bar = 1 mm. (E) Atherosclerotic lesion area in the thoracic aorta; N = 10 per group. AngI: Angiotensin I; GalNAc AGT ASO: N-acetylgalactosamine-conjugated antisense oligonucleotides targeting angiotensinogen; PBS: Phosphate-buffered saline.

A single dose of GalNAc AGT ASO reduced plasma AGT maximally within 3 days after the injection (Figure 3A). Therefore, when comparing GalNAc AGT ASO (5 mg/kg) and losartan, we began feeding the Western diet 1 week after the initiation of GalNAc AGT ASO (Figure 3B). GalNAc AGT ASO significantly reduced plasma AGT concentrations, while losartan had no effect (Figure 3C). A similar pattern was observed for hepatic AGT mRNA abundance (Figure 3D). As expected, both drugs increased plasma renin concentrations and renal renin mRNA abundance (Figure 4A and B), but the effects of GalNAc AGT ASO was greater than that of losartan. This coincided with the effect on systolic BP (Figure 4C), which was lowered more significantly by GalNAc AGT ASO. In contrast, both drugs equivalently reduced atherosclerotic lesion areas (Figure 4D and E).

**Figure 5.**
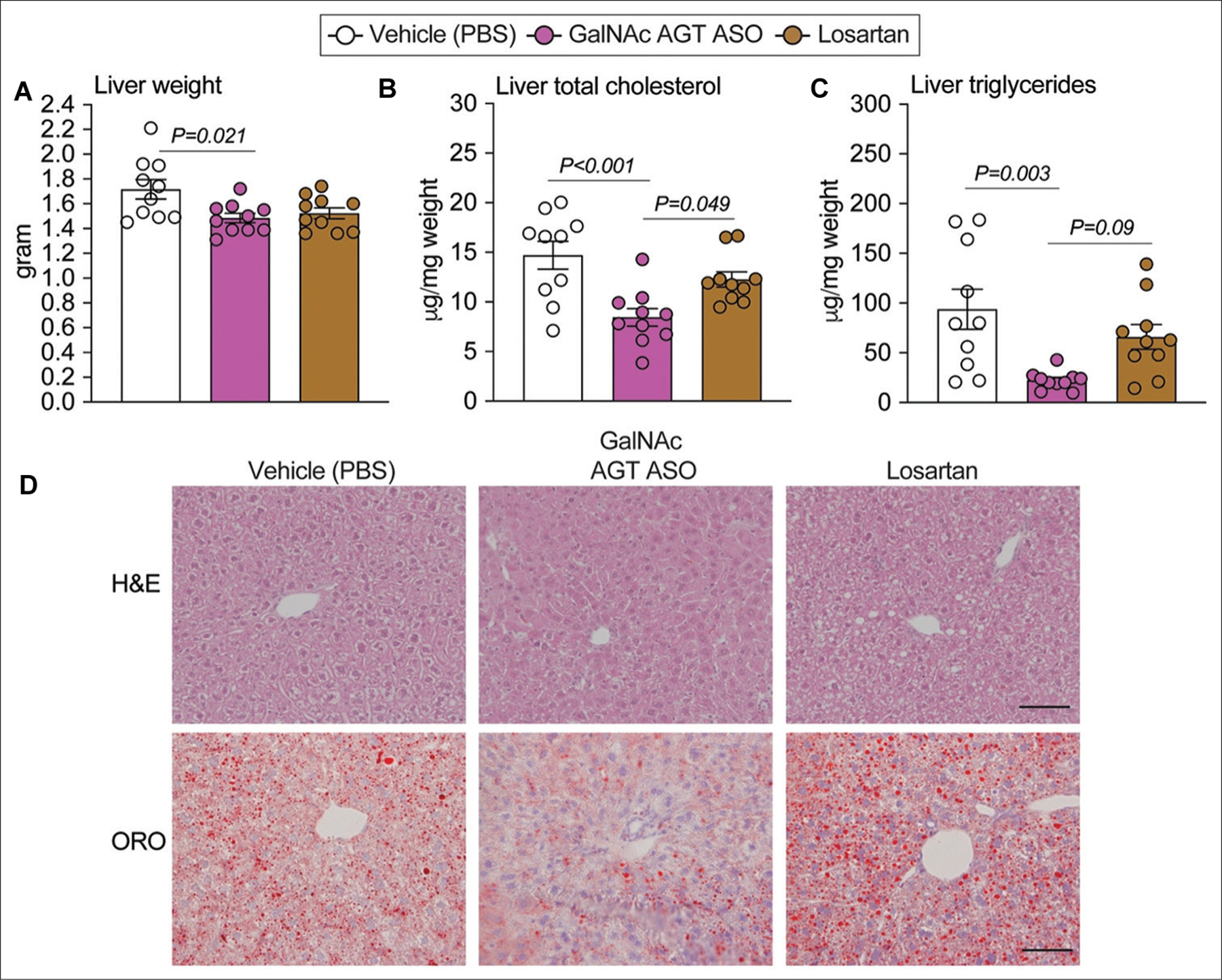
Effects of hepatocyte-specific antisense oligonucleotides of angiotensinogen versus losartan on liver steatosis. (A) Liver weight at termination. (B) Liver total cholesterol concentration (normalized by liver weight). (C) Liver triglycerides concentration (normalized by liver weight). (D) Hematoxylin and eosin (H and E) staining and Oil Red O (ORO) staining of liver sections; scale bar = 200 μm. N = 10 per group. GalNAc AGT ASO: N-acetylgalactosamine-conjugated antisense oligonucleotides targeting angiotensinogen; PBS: Phosphate-buffered saline.

Neither drug increased plasma ALT or AST concentrations (Supplementary File, Figure S1), indicating that GalNAc AGT ASO and losartan do not cause liver damage. Consistent with observations in the first study (Figure 2), GalNAc AGT ASO reduced liver weight and lipid accumulation in the liver (Figure 5). Compared to the vehicle group, losartan did not affect liver weight, nor reduced the total cholesterol, triglycerides, or the severity of neutral lipid accumulation in the liver (Figure 5).

## 4. Discussion

This study demonstrated that GalNAc AGT ASO reduced systolic BP and atherosclerosis in LDL receptor^−/−^ mice fed a Western diet. The effects of GalNAc AGT ASO 5 mg/kg were more pronounced in reducing systolic BP than losartan (15 mg/kg/day). This is likely because this ASO dose induced a greater degree of renin-angiotensin blockade, reflected by the higher renin increase observed after administering GalNAc AGT ASO compared to losartan. Of note, the effects of both drugs on atherosclerosis were not different. Although high BP is an independent risk factor for atherosclerosis, there is a growing body of evidence suggesting that BP *per se* does not contribute to atherosclerosis^[23]^. The different magnitudes of suppressing AGT versus blocking AT1 receptor on BP and atherosclerosis in this study support this notion. Overall, this study unequivocally shows that BP regulation and atherosclerosis in hypercholesterolemic LDL receptor^−/−^ mice are dependent on AngII generated from AGT of hepatic origin, thereby agreeing with the observations made in mice displaying genetic hepatocyte-specific AGT deficiency^[8,24,25]^.

Remarkably, GalNAc AGT ASO, but not losartan, reduced Western diet-induced liver steatosis, which was manifested as increased cholesterol and triglyceride content in the liver. This finding is consistent with our previous studies in hepAGT^−/−^ mice^[8,19]^. These effects occurred in a dose-independent manner and likely represent the direct consequence of lowering AGT, which occurred following GalNAc AGT ASO administration. Indeed, the expression of des(AngI)AGT induced liver steatosis in hepatocyte-specific AGT deficient mice fed a Western diet^[8]^. Given the liver-specific activity of GalNAc AGT ASO^[14]^, we would expect non-hepatic AGT to have no effect on liver steatosis.

It is important to note that mice, unlike humans, display a very high turnover of AGT, reflected by lower plasma AGT concentrations than in humans. Hence, the des(AngI)AGT/intact AGT ratio is higher in mice than in humans^[26]^. Although administration of losartan did not affect AGT and liver steatosis in the present study, earlier studies have reported suppression of liver steatosis in mice administered with RAS inhibitors^[27–29]^. This may reflect a different and larger degree of renin-angiotensin blockade, potentially lowering AGT^[30]^ and/or affecting the des(AngI)AGT/intact AGT ratio. The molecular mechanism underlying the direct effect of des(AngI) AGT is unknown. It may involve interference with the protein kinase B (Akt)/mammalian target of rapamycin (mTOR)/sterol responsive element-binding protein 1c (SREBP-1c) pathway^[19]^, which is associated with liver steatosis and suppressed in mice with hepatocyte-specific deletion of AGT. It is also unknown whether intact AGT exerts similar effects. Studying this would require the AAV-induced expression of intact AGT in hepAGT^−/−^ mice under complete renin-angiotensin blockade.

## 5. Conclusions

GalNAc AGT ASO not only reduces BP and atherosclerosis, but also improves diet-induced liver steatosis in mice. Future studies should investigate whether AGT suppression induces similar effects in humans and nonhuman primates.

## Supplementary Material

Supplementary File

## References

[1] Lu H, Cassis LA, Vander Kooi CW, , 2016, Structure and functions of angiotensinogen. Hypertens Res, 39: 492–500. 10.1038/hr.2016.17 26888118 PMC4935807

[2] Wu C, Lu H, Cassis LA, , 2011, Molecular and pathophysiological features of angiotensinogen: A mini review. N Am J Med Sci (Boston), 4: 183–190. 10.7156/v4i4p183 22389749 PMC3291105

[3] Daugherty A, Rateri DL, Lu H, , 2004, Hypercholesterolemia stimulates angiotensin peptide synthesis and contributes to atherosclerosis through the AT1A receptor. Circulation, 110: 3849–3857. 10.1161/01.CIR.0000150540.54220.C4 15596561

[4] Lu H, Rateri DL, Feldman DL, , 2008, Renin inhibition reduces hypercholesterolemia-induced atherosclerosis in mice. J Clin Invest, 118: 984–993. 10.1172/JCI32970 18274671 PMC2242618

[5] Lu H, Balakrishnan A, Howatt DA, , 2012, Comparative effects of different modes of renin angiotensin system inhibition on hypercholesterolaemia-induced atherosclerosis. Br J Pharmacol, 165: 2000–2008. 10.1111/j.1476-5381.2011.01712.x 22014125 PMC3372847

[6] Takata Y, Chu V, Collins AR, , 2005, Transcriptional repression of ATP-binding cassette transporter A1 gene in macrophages: A novel atherosclerotic effect of angiotensin II. Circ Res, 97: e88–e96. 10.1161/01.RES.0000190400.46267.7e 16224068

[7] Fusco FB, Gomes DJ, Bispo KC, , 2017, Low-sodium diet induces atherogenesis regardless of lowering blood pressure in hypertensive hyperlipidemic mice. PLoS One, 12: e0177086. 10.1371/journal.pone.0177086 28481921 PMC5421755

[8] Lu H, Wu C, Howatt DA, , 2016, Angiotensinogen exerts effects independent of angiotensin II. Arterioscler Thromb Vasc Biol, 36: 256–265. 10.1161/ATVBAHA.115.306740 26681751 PMC4732917

[9] Dickson ME, Sigmund CD, 2006, Genetic basis ofhypertension: Revisiting angiotensinogen. Hypertension, 48: 14–20. 10.1161/01.HYP.0000227932.13687.60 16754793

[10] Yiannikouris F, Karounos M, Charnigo R, , 2012, Adipocyte-specific deficiency of angiotensinogen decreases plasma angiotensinogen concentration and systolic blood pressure in mice. Am J Physiol Regul Integr Comp Physiol, 302: R244–R251. 10.1152/ajpregu.00323.2011 22071160 PMC3349391

[11] Yiannikouris F, Gupte M, Putnam K, , 2012, Adipocyte deficiency of angiotensinogen prevents obesity-induced hypertension in male mice. Hypertension, 60: 1524–1530. 10.1161/HYPERTENSIONAHA.112.192690 23108647 PMC3517298

[12] Matsusaka T, Niimura F, Shimizu A, , 2012, Liver angiotensinogen is the primary source of renal angiotensin II. J Am Soc Nephrol, 23: 1181–1189. 10.1681/ASN.2011121159 22518004 PMC3380650

[13] Rong J, Tao X, Lin Y, , 2021, Loss of hepatic angiotensinogen attenuates sepsis-induced myocardial dysfunction. Circ Res, 129: 547–564. 10.1161/CIRCRESAHA.120.318075 34238019

[14] Mullick AE, Yeh ST, Graham MJ, , 2017, Blood pressure lowering and safety improvements with liver angiotensinogen inhibition in models of hypertension and kidney injury. Hypertension, 70: 566–576. 10.1161/HYPERTENSIONAHA.117.09755 28716988

[15] Daugherty A, Rateri D, Lu H, , 2009, Measuring blood pressure in mice using volume pressure recording, a tail-cuff method. J Vis Exp, 27: 1291. 10.3791/1291 PMC2794298 19488026

[16] Daugherty A, Tall AR, Daemen MJ, , 2017, Recommendation on design, execution, and reporting of animal atherosclerosis studies: A scientific statement from the American heart association. Arterioscler Thromb Vasc Biol, 37: e131–e157. 10.1161/ATV.0000000000000062 28729366

[17] Chen H, Howatt DA, Franklin MK, , 2022, A mini-review on quantification of atherosclerosis in hypercholesterolemic mice. Glob Transl Med, 1: 72. 10.36922/gtm.v1i1.76 35925518 PMC9345319

[18] Kim S, Yang L, Kim S, , 2017, Targeting hepatic heparin-binding EGF-like growth factor (HB-EGF) induces anti-hyperlipidemia leading to reduction of angiotensin II-induced aneurysm development. PLoS One, 12: e0182566. 10.1371/journal.pone.0182566 28792970 PMC5549937

[19] Tao XR, Rong JB, Lu HS, , 2019, Angiotensinogen in hepatocytes contributes to Western diet-induced liver steatosis. J Lipid Res, 60: 1983–1995. 10.1194/jlr.M093252 31604805 PMC6889717

[20] Lu Z, Li Y, Li AJ, , 2022, Loss of GPR40 in LDL receptor-deficient mice exacerbates high-fat diet-induced hyperlipidemia and nonalcoholic steatohepatitis. PLoS One, 17: e0277251. 10.1371/journal.pone.0277251 36331958 PMC9635748

[21] Liu Y, Zhang Y, Zhu H, , 2022, Aucubin administration suppresses STING signaling and mitigated high-fat diet-induced atherosclerosis and steatohepatosis in LDL receptor deficient mice. Food Chem Toxicol, 169: 113422. 10.1016/j.fct.2022.113422 36108984

[22] de Jong LM, Zhang Z, den Hartog Y, , 2022, PRMT3 inhibitor SGC707 reduces triglyceride levels and induces pruritus in Western-type diet-fed LDL receptor knockout mice. Sci Rep, 12: 483. 10.1038/s41598-021-04524-w 35013582 PMC8748717

[23] Lu H, Cassis LA, Daugherty A, 2007, Atherosclerosis and arterial blood pressure in mice. Curr Drug Targets, 8: 1181–1189. 10.2174/138945007782403829 18045096

[24] Wu C, Xu Y, Lu H, , 2015, Cys18-Cys137 disulfide bond in mouse angiotensinogen does not affect AngII-dependent functions in vivo. Hypertension, 65: 800–805. 10.1161/HYPERTENSIONAHA.115.05166 25691624 PMC4359061

[25] Wu CH, Wu C, Howatt DA, , 2020, Two amino acids proximate to the renin cleavage site of human angiotensinogen do not affect blood pressure and atherosclerosis in mice. Arterioscler Thromb Vasc Biol, 40: 2108–2113. 10.1161/ATVBAHA.120.314048 32640904 PMC7483780

[26] Kukida M, Cai L, Ye D, , 2021, Renal angiotensinogen is predominantly liver derived in nonhuman primates. Arterioscler Thromb Vasc Biol, 41: 2851–2853. 10.1161/ATVBAHA.121.316590 34496634 PMC8551028

[27] Wang CH, Liu HM, Chang ZY, , 2021, Losartan prevents hepatic steatosis and macrophage polarization by inhibiting HIF-1α in a murine model of NAFLD. Int J Mol Sci, 22: 7841. 10.3390/ijms22157841 34360607 PMC8346090

[28] Stucchi P, Cano V, Ruiz-Gayo M, , 2009, Aliskiren reduces body-weight gain, adiposity and plasma leptin during diet-induced obesity. Br J Pharmacol, 158: 771–778. 10.1111/j.1476-5381.2009.00355.x 19694726 PMC2765596

[29] Premaratna SD, Manickam E, Begg DP, , 2012, Angiotensin-converting enzyme inhibition reverses diet-induced obesity, insulin resistance and inflammation in C57BL/6J mice. Int J Obes (Lond), 36: 233–243. 10.1038/ijo.2011.95 21556046

[30] Cruz-López EO, Ren L, Uijl E, , 2023, Blood pressure-independent renoprotective effects of small interference RNA targeting liver angiotensinogen in experimental diabetes. Br J Pharmacol, 180: 80–93. 10.1111/bph.15955 36106615 PMC10091936

