# Supplementary material for: Antisense oligonucleotides targeting hepatic angiotensinogen reduce atherosclerosis and liver steatosis in hypercholesterolemic mice
Source: Glob Transl Med. Author manuscript; Available in PMC 2023 Jun 8. (PMC10249463; doi:10.36922/gtm.288)
Supplement: Supplementary File [file NIHMS1875189-supplement-Supplementary_File.pdf]

ACCSCIENCE PUBLISHING
Global Translational Medicine

ORIGINAL RESEARCH ARTICLE

Antisense oligonucleotides targeting hepatic angiotensinogen reduce atherosclerosis and liver steatosis in hypercholesterolemic mice

Supplementary File

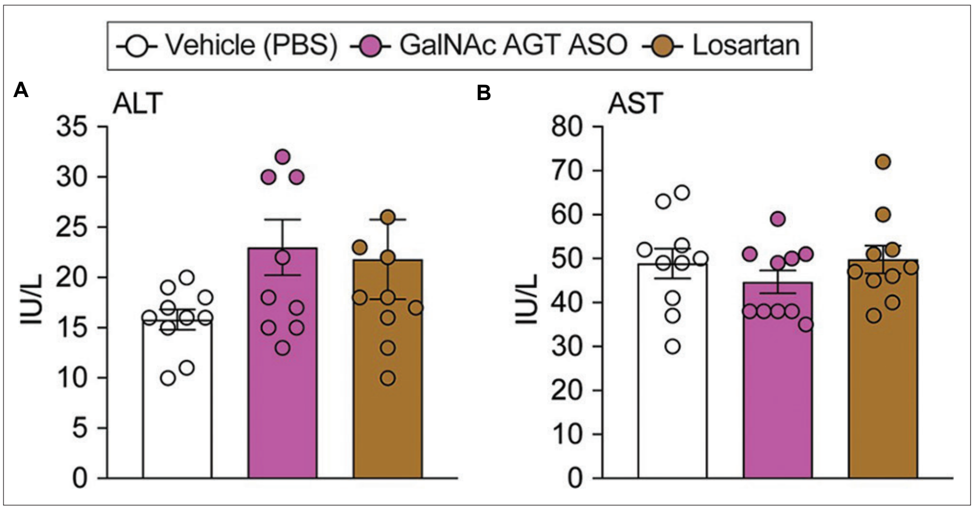

Figure S1. Plasma (A) alanine transaminase and (B) aspartate transaminase concentrations; N = 10 per group. *P*=0.14 for ALT and *P*=0.47 for AST were determined by one-way ANOVA.
GalNAc AGT ASO: N-acetylgalactosamine-conjugated antisense oligonucleotides targeting angiotensinogen; PBS: Phosphate-buffered saline.

Table S1. Animals used in *in vivo* studies

Species	Vendor or source	Background strain	Sex	Persistent ID/URL
Mouse	The Jackson Laboratory	LDL receptor -/-	Male	# 002207 https://www.jax.org/strain/002207
Mouse	The Jackson Laboratory	C57BL/6J	Male	# 000664 https://www.jax.org/strain/000664

Table S2. Design of Experiment 1 (Figures 1 and 2)

Group	Sex	Age (weeks)	Number (prior to experiment)	Number (after termination)
Vehicle (PBS)	Male	8	6	6
GalNAc AGT ASO 1 mg/kg	Male	8	10	10
GalNAc AGT ASO 2.5 mg/kg	Male	8	10	10
GalNAc AGT ASO 5 mg/kg	Male	8	8	8

GalNAc AGT ASO: N-acetylgalactosamine-conjugated antisense oligonucleotides targeting angiotensinogen; PBS: Phosphate-buffered saline

Table S3. Design of Experiment 2 (Figure 3A)

Group	Sex	Age (weeks)	Number (prior to experiment)	Number (after termination)
GalNAc AGT ASO 10 mg/kg	Male	8	5	5

GalNAc AGT ASO: N-acetylgalactosamine-conjugated antisense oligonucleotides targeting angiotensinogen

Table S4. Design of Experiment 3 (Figures 3B–5)

Group	Sex	Age (weeks)	Number (prior to experiment)	Number (after termination)
Vehicle (PBS)	Male	8	10	10
GalNAc AGT ASO 5 mg/kg	Male	8	10	10
Losartan 15 mg/kg/day	Male	8	10	10

GalNAc AGT ASO: N-acetylgalactosamine-conjugated antisense oligonucleotides targeting angiotensinogen; PBS: Phosphate-buffered saline

Table S5. Animal Research: Reporting of *In Vivo* Experiments (ARRIVE) guidelines checklist

Item	Description
Ethics	Approved by the University of Kentucky IACUC
Sex	Male only
Inclusion criteria (when mice were enrolled for experiments)	(1) Sex: Male<br>(2) Age: ~8 weeks old<br>(3) Body weight>or=20 g<br>(4) Mouse strain: $LDLR^{-/-}$ in C57BL/6J background
Exclusion criteria	(1) Body weight: <20 g (when mice were enrolled for experiments)<br>(2) Euthanasia prior to the endpoint due to any humane reason, including severe skin lesions, body weight loss>20% within 1 week, or requested by a veterinarian based on IACUC regulation
Sample size	Described in each figure legend
Power analysis	Not performed
Endpoint	Primary: atherosclerotic lesion size
Randomization	Study mice were randomly placed in cages by the DLAR staff and grouped randomly by an investigator
Blinding	All experimental data were verified by an independent investigator blinded to the study group information
Statistical analysis	Verified by a biostatistician, Dr. Yuriko Katsumata
Statistical method	Described in the Methods section
Data availability	All numerical data are available in the Excel File "Data for Figures." All raw data and analytical methods are available from the corresponding author upon appropriate request.

DLAR: Division of Laboratory Animal Resources; IACUC: Institutional Animal Care and Use Committee; LDLR: Low-density lipoprotein receptor

Table S6. Mouse housing conditions

Description	Mouse housing conditions	Note
Diet	Diet # 2918 or Diet # TD.88137 (Envigo)	*Ad libitum*
Water	Reverse osmosis (RO) water	*Ad libitum*
Bedding	Aspen hardwood chips, # 7090A, Harlan Teklad Global	
Enrichment	None	
Set temperature range	68 – 74°F	
Set humidity	50 – 60%	
Light cycle (light: dark)	14:10 h	
Specific-pathogen-free	Yes	
